# Evaluation of Functional Components of *Lactobacillus plantarum* AR495 on Ovariectomy-Induced Osteoporosis in Mice And RAW264.7 Cells

**DOI:** 10.3390/foods13193115

**Published:** 2024-09-29

**Authors:** Zheng Chen, Junlin Shao, Yijin Yang, Guangqiang Wang, Zhiqiang Xiong, Xin Song, Lianzhong Ai, Yongjun Xia, Beiwei Zhu

**Affiliations:** 1 School of Food Science and Technology, Dalian Polytechnic University, Dalian 116034, China; sky_527@163.com; 2 School of Health Science and Engineering, Shanghai Engineering Research Center of Food Microbiology, University of Shanghai for Science and Technology, Shanghai 200093, China; shaojunlin02@163.com (J.S.); soliaran@163.com (Y.Y.); 1015wanggq@163.com (G.W.); xiongzhiq@163.com (Z.X.); daohongxuan@126.com (X.S.); ailianzhong1@126.com (L.A.)

**Keywords:** *Lactobacillus plantarum*, osteoporosis, RANK/RANKL/OPG, functional component

## Abstract

Osteoporosis is a disease characterized by abnormal bone metabolism, where bone resorption outpaces bone formation. In this study, we investigated the key functional components of *Lactobacillus plantarum* AR495 in mitigating ovariectomy (OVX)-induced osteoporosis in mice. The results indicated that both Lactobacillus plantarum AR495 and its fermentation broth significantly reduced urinary calcium and deoxypyridinoline (DPD) levels in the mice. These interventions inhibited bone resorption and improved trabecular bone architecture by modulating the nuclear factor κB (RANK)/RANK ligand (RANKL)/osteoprotegerin (OPG) signaling pathway. Additionally, the L. plantarum AR495 and fermentation broth groups inhibited the RANKL/TRAF-6 and TLR4/MYD88 pathways, leading to enhanced bone metabolism, improved intestinal barrier function, and reduced intestinal inflammation. In vitro experiments revealed that AR495 fermentation supernatant fractions larger than 100 kDa and those between 50–100 kDa significantly decreased the activity of the osteoclast marker TRAP, regulated the expression of the TLR4/MYD88 pathway, and inhibited osteoclast formation, thereby alleviating the OVX-induced osteoporosis phenotype. These findings suggest that these components may be primary functional elements of *L. plantarum* AR495 in the treatment of osteoporosis.

## 1. Introduction

Osteoporosis is a systemic condition characterized by reduced bone density and mass, leading to increased bone fragility and a higher risk of fractures [1]. Bone is a dynamically renewing organ that continuously undergoes remodeling, shaping, and repair throughout its lifecycle. Osteoblast-mediated bone formation and osteoclast-mediated bone resorption are integral processes that maintain normal bone metabolic homeostasis. These processes operate synergistically in both temporal and spatial dimensions to regulate bone remodeling [2]. Disruptions in this balance, characterized by decreased bone formation and increased bone resorption, can lead to common metabolic disorders such as osteoporosis [3]. A significant risk factor for osteoporosis is the loss of estrogen during menopause, which primarily causes an imbalance between bone resorption and bone formation due to reduced estrogen levels [4].

Hormone replacement therapy (HRT) has historically been used as a primary intervention for the prevention of postmenopausal osteoporosis, showing observable benefits when initiated during early menopause. However, prolonged use of HRT is associated with an increased risk of breast cancer and cardiovascular disease [5]. In contrast, selective estrogen receptor modulators (SERMs), while not being estrogens themselves, bind to estrogen receptors and thereby exert a modulatory effect [6]. Bisphosphonates are among the most frequently employed conventional therapies for inhibiting osteoclast differentiation and maturation, as well as promoting their apoptosis; however, they may induce gastrointestinal disorders [7]. The majority of existing pharmacological treatments for osteoporosis are associated with adverse effects and limitations, underscoring the ongoing need for more effective interventions to enhance bone health and prevent the disease. In recent years, the conceptualization of the brain-gut axis, liver-gut axis, and bone-gut axis has underscored the pivotal role of gut microbiota in a myriad of diseases. Consequently, probiotics, which exert their effects directly within the gut and are associated with fewer side effects, hold significant promise for the amelioration of these conditions [8,9].

Probiotics are defined as “live microorganisms that are beneficial to the host when used in sufficient quantities” [10]. They are effective in alleviating allergies, inflammatory bowel disease, Alzheimer’s disease, and type II diabetes [11,12,13,14]. In the context of osteoporosis, specific strains of *Lactobacillus* and *Bifidobacterium* have been shown to enhance vitamin D absorption and inhibit osteoclast differentiation, thereby preventing bone loss induced by ovariectomy in murine models [15,16,17]. Notably, *Lactobacillus rhamnosus* ATCC PTA 6475 has been demonstrated to mitigate bone resorption and enhance bone metabolism through the inhibition of TNF-α production [18]. Similarly, the administration of *Lactobacillus acidophilus* in ovariectomized mice has been shown to improve the microarchitecture of both trabecular and cortical bone, in addition to increasing bone mineral density and heterogeneity [19]. Various treatments and components of probiotics have been identified to possess functional properties that contribute to these beneficial effects. Research indicates that the bioactive components of probiotics encompass cell wall fractions, surface proteins, nucleic acids, organic acids, short-chain fatty acids, antimicrobial proteins, and other soluble factors that are not easily identifiable [20]. Oral administration of pasteurized, inactivated Ackermann’s bacteria has been shown to lower total cholesterol levels, reduce body weight, and decrease fat accumulation [21]. Additionally, the cell wall proteins of *Lactobacillus* species can inhibit pathogenic organisms by adhering to lymph nodes and inducing a Th1-type immune response [20]. Certain metabolites produced by probiotics, including secreted proteins and organic acids, have been shown to enhance the integrity of the intestinal epithelial barrier. This is achieved through the promotion of mucus secretion from goblet cells and the increased production of antimicrobial peptides [21]. In addition, *Lactobacillus rhamnosus* GG (LGG) culture supernatant also protects the intestinal barrier in neonatal rats [22].

In a previous investigation, *L. plantarum* AR495 (AR495), isolated from Chinese rice wine lees, was shown to enhance bone calcium content in female mice and effectively mitigate osteoporosis symptoms [23]. The objective of the current study was to delve deeper into the specific components and mechanisms by which AR495 alleviates osteoporosis. Through a series of in vivo and ex vivo experiments, we observed that both AR495 and its fermentation supernatant improved bone trabecular structure, enhanced intestinal barrier function, and maintained balanced bone metabolism. Additionally, both the >100 kDa and 50–100 kDa fractions of the fermentation supernatant were found to attenuate OVX-induced osteoporosis. This study not only provides insights into the active ingredients involved in probiotics’ role but also offers further examples of how probiotics can ameliorate osteoporosis.

## 2. Materials and Methods

### 2.1. Chemicals and Reagents

A creatinine (Cr) assay kit was purchased from Nanjing Jianjian Bioengineering Institute (Nanjing, China). A calcium assay kit (o-cresolphthalein complexone method) was purchased from Zhongsheng Beizhong Biotechnology Co., Ltd. (Beijing, China). A PrimeScript RT reagent kit was purchased from Takara Biomedical Technology Co., Ltd. (Beijing, China). Hieff™ qPCR SYBR^®^ Green Master Mix was purchased from Yeasen Biotechnology Co., Ltd. (Shanghai, China). Tumor necrosis factor α (TNF-α), interleukin (IL)-1β, and DPD enzyme-linked immunosorbent assay (ELISA) kits were purchased from Shanghai Tongwei Biotechnology Co., Ltd. (Shanghai, China). RANKL was purchased from Thermo Fisher Scientific (Waltham, MA, USA). A CCK8 test kit, anti-tartrate acid phosphatase (TRAP), and a BCA kit were purchased from Biyuntian Biotechnology Co., Ltd. (Shanghai, China). A RAW 264.7 cell line was provided by the Cell Resource Centre of Shanghai Institutes for Biological Sciences, Chinese Academy of Sciences (SIBS). *Lactobacillus plantarum* AR495 was from the Institute of Food Biotechnology, the University of Shanghai for Science and Technology. A BioTek ELx800 Enzyme Labeling Instrument was purchased from BioTek Instruments, Inc. (Biotek, VT, USA).

### 2.2. Different Treatments of Strains

*Lactobacillus plantarum* AR495 was subjected to centrifugation following liquid culture in Modified MRs Medium Base (MRs). The resulting supernatant constituted the fermentation broth (fermentation broth group), while the pellet was resuspended in PBS to obtain live bacteria (live bacteria group). The resuspended bacteria were then subjected to a boiling water bath to achieve heat inactivation, resulting in dead bacteria (dead bacteria group). Additionally, the resuspended bacteria were sonicated for 45 min using an ultrasonic crusher and subsequently centrifuged, and the supernatant was the intracellular material (Intra-cellular material group); the sediment was taken to obtain the cell wall (cell wall group). The non-bacterial components were decontaminated by filtration through a 0.44 μm filter. The fermentation solution group utilized an MRs medium as a control to mitigate the interference from the medium (MRs group). *Lactobacillus plantarum* AR495 (preservation number CGMCC No. 14004) was sourced from the laboratory’s bacterial repository, and stored at −80 °C.

### 2.3. Different Treatments of AR495 Fermentation Broths

Take the fermentation broth and add it to a 100 kDa ultrafiltration centrifuge tube, centrifuge it at 4500 rpm 4 °C for 20 min, and then the concentrated fraction is fixed to 15 mL with sterile water, centrifuge it at 4500 rpm 4 °C for 20 min, and then wash it 3 times, and then collect the concentrated broth, which is fractioned with a molecular weight greater than 100 kDa; collect the remaining liquid with a molecular weight fraction greater than 100 kDa removed, and add it to a 50 kDa ultrafiltration centrifuge tube, and then the same operation is carried out to collect the fractions of 50–100 kDa and 10–50 kDa sizes, and meanwhile, the fraction with a molecular weight of less than 10 kDa is also collected.

### 2.4. Experimental Animals and Design

**Figure 1:**
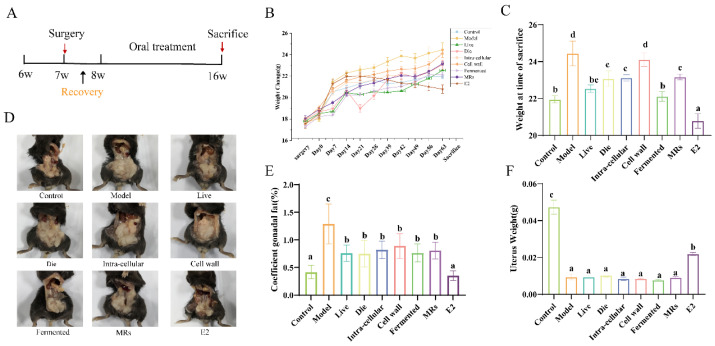
Effect of different treatments of AR495 on body weight and gonadal adiposity. (**A**) Experimental flow chart; (**B**) Weight change; (**C**) Weight at time of sacrifice t; (**D**) Gonadal fat accumulation in mice; (**E**) Coefficient gonadal; (**F**) Uterus Weight. All data are expressed as mean ± SD and were analyzed by one-way ANOVA and Duncan’s multiple comparison test, n = 10. Significant differences (*p* < 0.05) are indicated with different letters (a, b, c, and d).

Eight-week-old female C57BL/6 mice were procured from Shanghai Jiesijie Laboratory Animal Co., Ltd., Shanghai, China. The mice were maintained under standard conditions, at a temperature of 25 ± 2 °C, a humidity of 50 ± 5%, and a 12-h light/dark cycle. The mice were provided with low-calcium and low-phosphorus food and sterile water. After one week of acclimatization, mice were randomly divided into 9 groups (n = 10): control, model, live, dead, intra-cellular, cell wall, fermentation broth, MRs, and positive control estradiol-treated (E2). The control and model groups received daily gavage with sterile water. The bacterial solution was administered at a concentration of 1 × 10^9^ CFU/mL (10 mL/kg per day) in the AR495 live and dead groups. The positive control group, treated with estradiol (E2), received a daily gavage dose of 10 mg/kg based on body weight for 9 weeks. All experimental treatments were administered via gavage, with specific dosages detailed in Table 1. Mice were weighed weekly, and urine samples were collected after the final gavage. The experimental procedure is illustrated in Figure 1A.

Mice were anesthetized with an intraperitoneal injection of 0.7% sodium pentobarbital at a dosage of 10 mg/kg of body weight. After anesthesia was induced and under aseptic conditions, incisions were made in the dorsal skin and muscularis propria. In the control group, only wounds were created and subsequently sutured. For the experimental group, the uterus was isolated, and the ovaries were excised, except in the sham-operated group. The wounds were then cleaned, and the muscularis propria and skin were sutured together using surgical forceps. After the procedure, the mice were returned to their feeding cages upon regaining consciousness.

### 2.5. Micro-Computed Tomography (μCT)

The bone parameters of the mouse femurs were observed by micro-CT scanning (Lecia, Wetzlar, Germany) to obtain the three-dimensional stereo structure of trabecular and cortical bone as well as the corresponding 3D statistical data and morphological parameters.

### 2.6. Measurements of Bone Turnover Markers

Use the ELISA kits in strict accordance with the Deoxypyridinoline (DPD). A Creatinine (Cr) Assay Kit and Calcium Assay Kit (o-cresolphthalein complexone method) were used strictly according to the instructions.

### 2.7. ELISA Assay

The expression of cellular inflammatory factors was measured using an ELISA kit (Tongwei, Shanghai, China) according to the manufacturer’s instructions. The OD value of the cytokine plate was measured with a microplate reader. 

### 2.8. RANKL-Induced Cell Differentiation

Cells were spread in well plates at 1 × 10^4^ cells/mL and induced by adding medium containing 50 ng/mL RANKL when the cells were attached to the wall.

### 2.9. Cell Viability, Counting, and Cell Morphology 

Cellular activity was assessed using the CCK8 kit. Specifically, 10 μL of CCK8 reagent was added to each well containing cultured RAW264.7 cells. After an incubation period of 1 h at 37 °C in a light-protected incubator, absorbance measurements were taken. Additionally, cells were stained according to the protocol provided by the Anti-Tartrate Acid Phosphatase Stain Kit. Observations were conducted using a fluorescence-inverted microscope, and images were captured. For each treatment condition, eight random fields of view were photographed, and multinucleated osteoblasts—defined as cells containing more than two nuclei—were counted.

### 2.10. Gene Expression

Total RNA was extracted from colon, tibia, and RAW264.7 cell samples (Sangon Biotech, Shanghai, China), and integrity was determined by agarose gel electrophoresis using a Gel Doc XR+ system (Bio-Rad, Hercules, CA, USA). RNA was reverse transcribed to cDNA according to the kit requirements and then performed at a uniform concentration for real-time quantitative polymerase chain reaction (RT-qPCR). HPRT1 was used as the reference control gene. According to the 2^−∆∆CT^, the mRNA relative expression levels were calculated. All the primers were synthesized by Sangon Biotech (Shanghai, China), and the sequences are listed in Table 2.

### 2.11. Statistical Analysis

All data were expressed as mean ± SD and analyzed by one-way analysis of variance (ANOVA), followed by Duncan’s test for multiple comparisons. Statistical analysis was carried out using SPSS v.22 (IBM Co., New York, NY, USA). *p*-values less than 0.05 were considered statistically significant.

## 3. Results

### 3.1. Effect of Different Treatments of AR495 on Body Weight and Gonadal Adiposity

The experimental flowchart is shown in Figure 1A. All ovariectomized (OVX) mice exhibited an increase in body weight relative to the control group (Figure 1B). By day 63, the body weights of mice in the live bacteria, fermentation solution, and E2 treatment groups were not significantly different from those of the control group (Figure 1C, *p* > 0.05). Estrogen deficiency influences the centripetal distribution of gonadal fat. As illustrated in Figure 1D,E, gonadal fat was significantly reduced in all groups, except for the sham operation group, when compared to the model group (*p* < 0.05). The success of OVX was confirmed by the reduced uterine weight in all OVX mice compared to the control group mice (Figure 1F, *p* < 0.05). After a 9-week gavage period, no significant differences in uterine mass were observed among the groups, except for the control and E2 groups, further supporting the success of the OVX procedure (Figure 1F, *p* > 0.05).

### 3.2. Effect of Different Treatments of AR495 on Bone Morphology and Bone Conversion Markers

**Figure 2:**
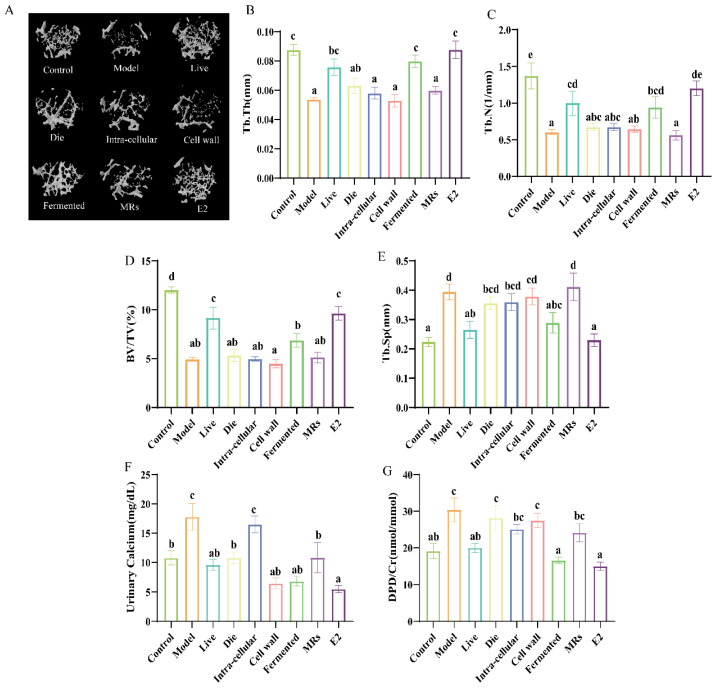
Effect of different treatments of AR495 on bone morphology and bone conversion markers. (**A**) Images of representative μCT reconstructions of examined trabecular bone volume; (**B**) Trabecular thickness (Tb.Th); (**C**) Trabecular number (Tb.N); (**D**) Percentage of tissue volume (BV/TV); (**E**) trabecular space (Tb.Sp); (**F**) calcium in urine; (**G**) bone resorptive biomarker deoxypyridinoline (DPD) in urine. All data are expressed as mean ± SD and analyzed by one-way ANOVA and Duncan’s multiple comparison test, n = 10. Significant differences (*p* < 0.05) are indicated with different letters (a, b, c, d, and e).

Micro-computed tomography (µCT) analysis of the femur (Figure 2A) revealed that solvent-treated ovariectomized (OVX) mice exhibited increased osteolytic activity and compromised bone microarchitecture. This included changes in parameters such as bone volume fraction (BV/TV), trabecular separation (Tb.Sp), trabecular number (Tb.N), and trabecular thickness (Tb.Th) compared to the control group (Figure 2B–E). Conversely, the groups treated with live bacteria and fermentation solution demonstrated a reversal of trabecular damage, enhancement of bone microstructure, a reduction in the BV/TV ratio, and a rebalancing of bone metabolism in OVX mice (Figure 2A–E, *p* < 0.05). With daily gavage of AR495 live bacteria or fermentation solution for 9 weeks showed a significant reversal of most of these altered parameters of bone metabolism or bone microarchitecture (Figure 2A–E, *p* < 0.05), and the effects were more stable.

Calcium levels serve as indicators of changes in calcium metabolism and bone metabolism, while the DPD/Cr ratio provides an objective measure of bone resorption. The administration of AR495 via gavage led to a significant reduction in urinary calcium levels across the live, dead, cell wall, fermentation broth, and E2 groups (Figure 2F, *p* < 0.05). However, despite the observed reduction in urinary calcium levels in the dead bacteria and cell wall groups, these groups did not exhibit a significant decrease in the DPD/Cr ratio (Figure 2G, *p* > 0.05). Notably, only the live bacteria and fermentation broth groups demonstrated a significant reduction in the DPD/Cr ratio (Figure 2G, *p* < 0.05).

### 3.3. Effect of Different Treatments of AR495 on the Expression of Pro-Osteoclast-Related Factors

**Figure 3:**
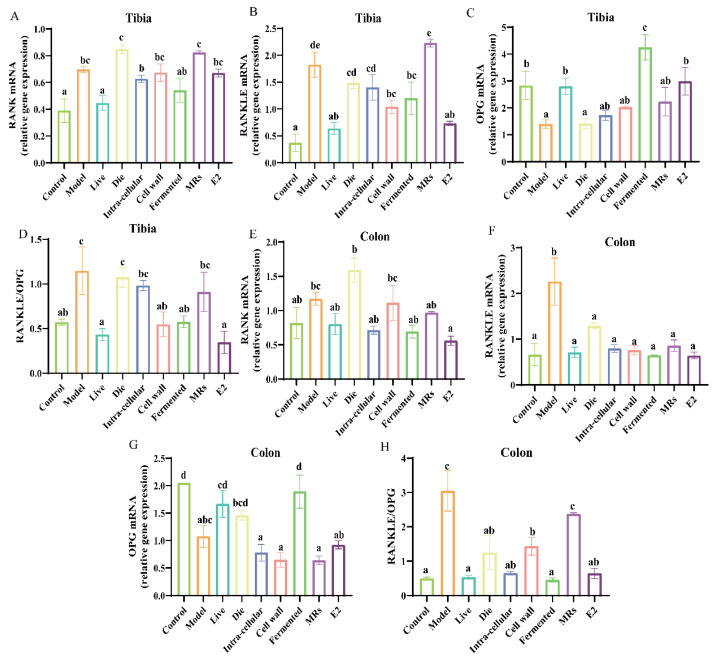
Effect of different treatments of AR495 on the expression of osteoclast-associated factors in tibia and colon. (**A**−**C**) The relative gene expression of RANK, RANKLE, and OPG in the tibia; (**D**) The ratio of receptor activator of nuclear factor-κ B ligand to osteoprotegerin (RANKL/OPG) in the tibia; (**E**−**G**) The relative gene expression of RANK, RANKLE, and OPG in the colon; (**H**) The ratio of receptor activator of nuclear factor-κ B ligand to osteoprotegerin (RANKL/OPG) in the colon. All data are expressed as mean ± SD and analyzed by one-way ANOVA and Duncan’s multiple comparison test, n = 10. Significant differences (*p* < 0.05) are indicated with different letters (a, b, c, and d).

The OPG/RANKL/RANK system is critical in the pathogenesis of postmenopausal osteoporosis. In the tibia of the model group, the expression level of OPG was significantly decreased, while the expression levels of RANK and RANKL, as well as the RANKL/OPG ratio, were significantly increased (Figure 3A–D, *p* < 0.05). Conversely, the AR495 live bacterial group and its fermentation broth significantly reduced the expression level of RANKL, increased the expression level of OPG, and decreased the RANKL/OPG ratio (Figure 3A–D, *p* < 0.05).

In the colon, our findings indicate that, contrary to inhibiting RANK expression, the group treated with dead bacteria exhibited a significant increase in RANK expression compared to the model group (Figure 3E, *p* < 0.05). Furthermore, all components of AR495 markedly reduced the expression levels of RANKL relative to the model group (Figure 3F, *p* < 0.05). The fermentation broth group exhibited a significant upregulation in the expression of OPG (Figure 3G, *p* < 0.05). Furthermore, the ratio of RANKL to OPG indicated that the live bacterial group, the intracellular material group, and the fermentation solution group were capable of modulating the expression of osteoclast-associated factors, with no statistically significant differences observed compared to the control group (Figure 3F, *p* > 0.05). However, concerning the regulation of the OPG/RANKL/RANK system, the dead bacteria group, cell wall group, MRs group, and intra-cellular material group demonstrated lower efficacy compared to the live bacteria group and fermentation broth group.

### 3.4. Effect of Different Treatments of AR495 on Intestinal Permeability in Mice

**Figure 4:**
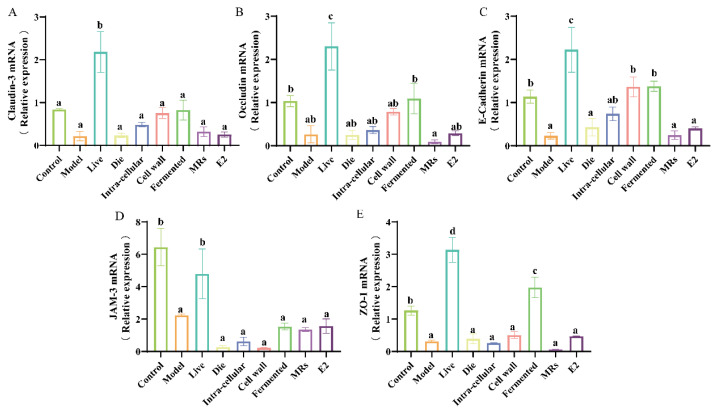
Effect of different treatments of AR495 on intestinal permeability in mice. (**A**) The relative gene expression of Claudin-3 in the colon. (**B**) The relative gene expression of Occludin in the colon; (**C**) The relative gene expression of E-Cadhcrin in the colon; (**D**) The relative gene expression of JAM-3 in the colon; (**E**) The relative gene expression of ZO-1 in the colon. All data are expressed as mean ± SD and analyzed by one-way ANOVA and Duncan’s multiple comparison test, n = 10. Significant differences (*p* < 0.05) are indicated with different letters (a, b, c, and d).

Estrogen deficiency compromises the integrity of the intestinal epithelial structure, allowing harmful substances to infiltrate the body’s internal environment and subsequently triggering an inflammatory response. The live bacterial group effectively increased the gene expression levels of intestinal tight junction proteins (Figure 4A–E, *p* < 0.05). In addition to JAM-3, the fermentation broth fraction also significantly increased the gene expression levels of Claudin-3, Occludin, E-Cadherin, and ZO-1 (Figure 4A,B,C,E, *p* < 0.05). The additional treatment groups receiving AR495 exhibited no statistically significant impact on intestinal tight junction proteins (Figure 4A–E, *p* > 0.05). Furthermore, our findings indicate that while the E2 group significantly ameliorated various osteoporosis-related parameters, it did not enhance intestinal permeability (Figure 4A–E, *p* > 0.05). This phenomenon may be attributed to specific active compounds present in live bacteria or fermentation solutions, which serve to fortify the intestinal barrier, modulate the intestinal microbiota, and suppress the proliferation of pathogenic bacteria.

### 3.5. Effects of Different Treatments of AR495 on Inflammatory Factors and Osteoclast Differentiation Pathways

**Figure 5:**
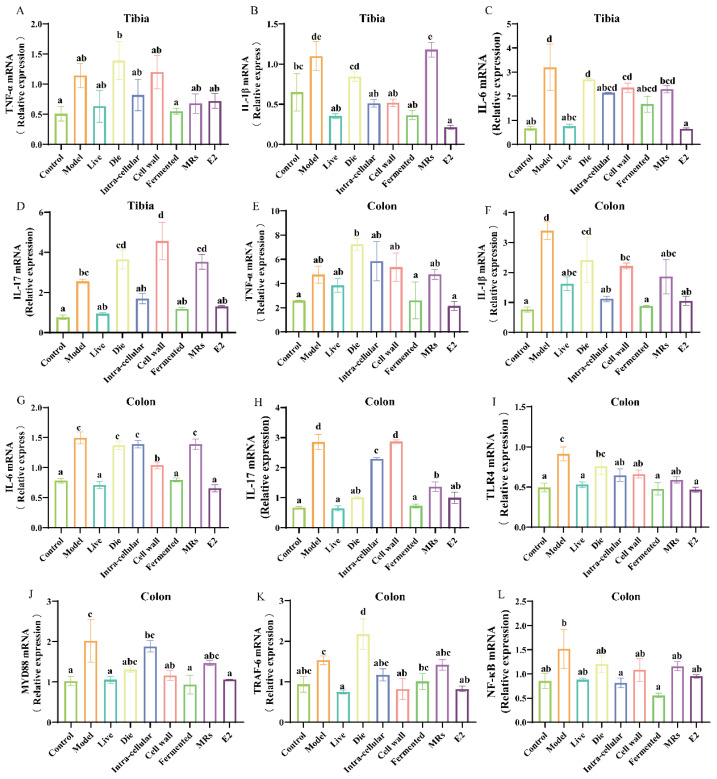
Effect of different treatments of AR495 on inflammatory factors and bone metabolic pathways. (**A–D**) The relative gene expression of TNF-α, IL-1β, IL-6, and IL-17 in the tibia; (**E–H**) The relative gene expression of TNF-α, IL-1β, IL-6, and IL-17 in the colon; (**I–L**) The relative gene expression of TLR4, MYD88, TRAF-6, and NF-κB in the colon. All data are expressed as mean ± SD and analyzed by one-way ANOVA and Duncan’s multiple comparison test, n = 10. Significant differences (*p* < 0.05) are indicated with different letters (a, b, c, and d).

The weakening of the intestinal barrier triggers an immune response and increases the expression of pro-inflammatory factors. Notably, elevated levels of these pro-inflammatory factors are associated with the formation and differentiation of osteoclasts. In the tibia, the presence of live bacteria, intracellular material, and the fermentation broth of AR495 significantly inhibited the gene expression of TNF-α, IL-1β, IL-6, and IL-17 (Figure 5A–D, *p* < 0.05). Similarly, in the colon, live bacteria and AR495 fermentation broth demonstrated a comparable effect in reducing pro-inflammatory factor levels (Figure 5E–H, *p* < 0.05). Overall, the live bacteria and fermentation broth groups were able to exert their anti-inflammatory effects more consistently, although other treatments with AR495 may also reduce the expression of certain pro-inflammatory factors. Furthermore, our findings indicate that the fermentation broth group exhibited a superior ability to inhibit the expression of inflammatory factors in the colon.

The RANKL/TRAF-6 and TLR4/MYD88/NF-κB pathways both induced osteoclast differentiation and elicited an inflammatory response. Notably, the expression levels of TLR4, MYD88, and TRAF-6 proteins were significantly diminished in the groups treated with either live bacteria or fermentation broth, as well as in the E2 group (Figure 5I–K, *p* < 0.05). Furthermore, the fermentation broth group demonstrated a superior capacity to reduce the gene expression level of NF-κB protein compared to the live bacteria group (Figure 5L, *p* < 0.05). In terms of inflammation-related pathways, both the live bacteria group and the fermentation broth group demonstrated significant inhibitory effects. This suggests that the live bacteria and fermentation broth components of AR495 have considerable potential for ameliorating osteoporosis.

### 3.6. Effects of Different Molecular Weight Fractions of AR495 Fermentation Broth on Bone Metabolism and Inflammation in RAW264.7 Cells

**Figure 6:**
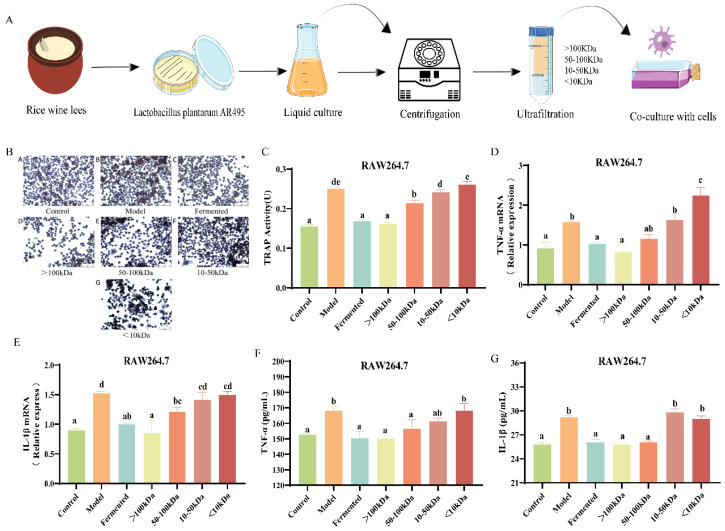
Effects of different molecular weight fractions of AR495 fermentation broth on bone metabolism and inflammation in RAW264.7 cells. (**A**) Co-culture of different fractions of fermentation broth with cells; (**B**) Osteoclast morphology observation microscopic findings (×200); (**C**) TRAP activity after co-culture of RAW264.7 and AR495 with different molecular weight fractions; (**D**,**E**) The relative gene expression of the TNF-α and IL-1β; (**F**,**G**) The protein expression of the TNF-α and IL-1β. All data are expressed as mean ± SD and analyzed by one-way ANOVA and Duncan’s multiple comparison test, n = 10. Significant differences (*p* < 0.05) are indicated with different letters (a, b, c, d, and e).

The flow of the cellular experiment is shown in Figure 6A. Except for the <10 kDa fraction, there was no significant difference in cell viability after the co-culture of each fraction in the fermentation broth with cells compared to the model group (Table S1, *p* < 0.05). Tartrate-resistant acid phosphatase (TRAP) staining revealed a significant increase in both the number and size of osteoclasts following RANKL induction. In contrast, the cell morphology in the blank group remained predominantly mononuclear and rounded, exhibiting a dark purple stain (Figure 6B). Compared to the model group, the number of osteoclasts in the fermentation broth groups (>100 kDa, 50–100 kDa, and 10–50 kDa) was significantly reduced (Table S2, *p* < 0.05). The fraction of the fermentation broth with a molecular weight greater than 100 kDa exhibited a more pronounced inhibitory effect on osteoclast differentiation (Table S2, *p* < 0.05). Similarly, TRAP activity was significantly reduced in the fermentation broth group, the >100 kDa group, and the 50–100 kDa group (Figure 6C, *p* < 0.05), especially in the >100 kDa fraction of the fermentation broth group (Figure 6C, *p* > 0.05). Upon induction of RAW264.7 cells with RANKL, the expression levels of TNF-α and IL-1β were significantly elevated in the model group (Figure 6D,E, *p* < 0.05). Conversely, treatment with the fermentation broth, specifically the >100 kDa and 50–100 kDa molecular weight fractions, mitigated this increase (Figure 6 D,E, *p* < 0.05). Notably, the >100 kDa molecular weight fraction demonstrated a more pronounced effect in reducing the protein expression of TNF-α and IL-1β (Figure 6F,G, *p* < 0.05).

### 3.7. Effect of Different Molecular Weight Fractions of AR495 Fermentation Broth on RAW264.7 Pathway Proteins

**Figure 7:**
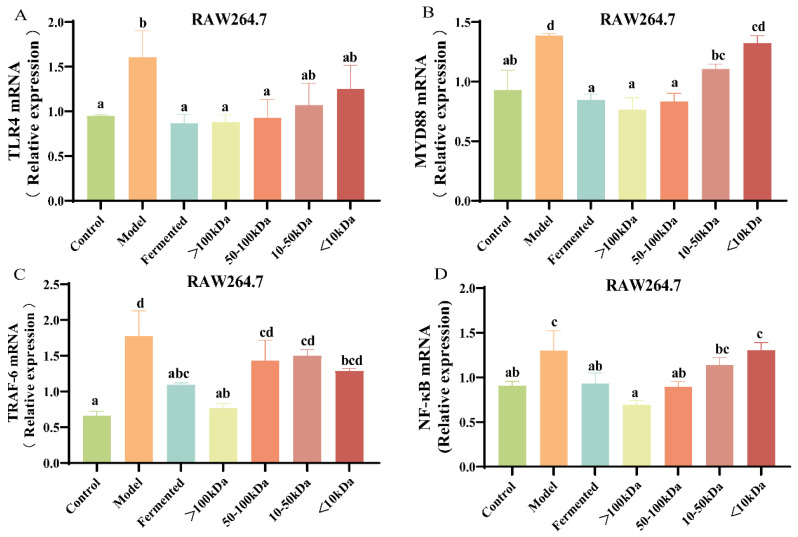
Effect of different molecular weight fractions of AR495 fermentation broth on RAW264.7 pathway proteins. (**A**–**D**) The relative gene expression of TLR4, MYD88, TRAF-6, and NF-κB. All data are expressed as mean ± SD and analyzed by one-way ANOVA and Duncan’s multiple comparison test, n = 10. Significant differences (*p* < 0.05) are indicated with different letters (a, b, c, and d).

We subsequently investigated whether the constituents of the fermentation broth influenced bone metabolism by modulating the RANKL/TRAF-6 or TLR4/MYD88/NF-κB pathways. Compared to the control group, RAW264.7 cells exhibited a significant upregulation in the gene expression levels of TLR4, MYD88, TRAF6, and NF-κB following RANKL induction (Figure 7A–D, *p* < 0.05). In comparison to the model group, the >100 kDa fraction completely reversed these effects (Figure 7A–D, *p* < 0.05). The 50–100 kDa fraction significantly reduced the relative gene expression levels of TLR4, MYD88, and NF-κB signaling proteins (Figure 7A,B,D, *p* < 0.05). However, it did not exert a statistically significant effect on the relative gene expression levels of TRAF-6 (Figure 7C, *p* > 0.05). This suggests that substances greater than 100 kDa in the fermentation broth may balance bone metabolism by inhibiting pathways that induce osteoclast formation. In contrast, the 50–100 kDa component may not directly influence bone loss through the osteoclast formation pathway. However, it is speculated that both the >100 kDa and 50–100 kDa fractions may have some inhibitory effect on osteoclast differentiation.

## 4. Discussion

This study aimed to isolate and investigate the functional components of AR495 in alleviating osteoporosis by evaluating the alleviating effects of live bacteria, dead bacteria, intracellular lysates, cell wall, fermentation broth, blank MRs group, and E2 group on the osteoporosis phenotype. Ultimately, we determined that AR495 and the fermentation broth group can balance bone metabolism by inhibiting the RANK/RANKL/OPG system, as well as the RANKL/TRAF-6 and TLR4/MYD88 pathways, thereby alleviating osteoporosis. It was concluded that the main active components in OVX may be proteins or polysaccharides, with the fraction above 100 kDa showing a more significant effect on osteoporosis.

### 4.1. The Relationship between Osteoporosis and the Gut 

Osteoporosis is classified as primary osteoporosis and secondary osteoporosis [24]. Primary osteoporosis is divided into two types: one related to estrogen deficiency during menopause, called postmenopausal osteoporosis, and the other related to calcium deficiency and bone aging, called senile osteoporosis [25]. In addition, disease, lifestyle, genetic disorders, medication, and other therapeutic interventions may also cause secondary osteoporosis [26]. Osteoporosis is also closely related to imbalances in the body’s immune system and the composition of the intestinal flora [27]. Intestinal flora can induce osteoclast differentiation and value-addition and exacerbate bone loss by inhibiting the TH17 cell ratio, as well as Th1, Th2, and Treg cell differentiation [27,28]. However, estrogen deficiency affects the intestinal barrier function, resulting in gut microbial dysbiosis [29]. In other words, there may be bidirectional communication between estrogen and the gut microbiota, and the two factors may influence each other [30]. Reports from the WHO indicate that the prevalence of osteoporosis in women is twice that in men in many countries. Contributing factors include a shorter time to bone maturation (peak bone mass) in women, a faster rate of bone loss after menopause compared to men, and common symptoms such as pain, susceptibility to falls, and fractures [31]. Osteoporosis as a “silent killer” has become a major problem for patients due to the side effects caused by the long-term use of conventional therapeutic drugs. Therefore, the search for safe and reliable active ingredients has become a research priority. More and more studies have demonstrated that probiotics have the potential to alleviate various diseases, but further research is needed to investigate the alleviation mechanism and active ingredients.

### 4.2. AR495 Alleviates Osteoporosis by Regulating the RANK/RANKL/OPG Signaling Pathway

Coordination between osteoblasts, which form bone, and osteoclasts, which dissolve and resorb bone, is an important cellular basis for the maintenance of bone function and homeostasis [32]. The RANK/RANKL/OPG signaling pathway plays an important role in the regulation of osteoclast differentiation [33]. RANK, a member of the TNF suprareceptor family, promotes the proliferation and differentiation of osteoclasts and enhances bone resorption activity [34]. RANKL is produced by neutrophils and osteoblasts in the bone marrow, and RANKL expression promotes the differentiation of osteoclast precursors into mature osteoblasts. However, RANKL binds to RANK and will promote osteoclastogenesis [23]. OPG is a soluble protein that blocks osteoclast formation in vitro and bone resorption in vivo. OPG competitively binds to RANKL and acts as a decoy receptor by blocking the binding of RANKL to its cytosolic receptor RANK, thereby reducing bone resorption [1]. In addition, an elevated RANKL/OPG ratio in bone marrow implies enhanced bone resorption [35]. In our study, we found that both in the tibia and the colon, the live bacteria and the fermentation broth groups were able to significantly reduce the RANKL/OPG ratio, leading to a decrease in bone resorption and thus a reduction in osteoporosis. Meanwhile, pro-inflammatory cytokines also affect the RANKL/OPG ratio, including TNF-α, IL-1, IL-1β, IL-6, and IL-17 [23]. The inhibition of IL-1 or TNF-α has been demonstrated to prevent the onset of osteoporosis in ovariectomized mice [36]. Conversely, IL-1, IL-17, and TNF-α have been shown to directly enhance RANKL production, while IL-6 promotes the production of IL-1 and IL-17 [23]. Our findings indicate that, in both the colon and tibia, the live, fermented solution of AR495 more consistently suppressed the relative expression of genes encoding TNF-α, IL-1β, and IL-6 compared to IL-17, thereby directly inhibiting RANKL production and osteoclastogenesis. While the intracellular material also reduced the expression of pro-inflammatory factors in the tibia, its effect was not significant in the colon. Similarly, the other fractions did not demonstrate the same consistency in effect as the live bacteria and fermentation broth groups.

### 4.3. Effects of Different Molecular Weight Fractions of AR495 Fermentation Broth on Bone Metabolism and Inflammation and Protein Pathway in RAW264.7 Cells 

RANKL binding to RANK triggers TRAF-6 and subsequently activates the TRAF-6-NF-κB signaling pathway [37]. In addition, the TLR4/MYD88/NF-κB pathway plays an important role in immunoinflammation and is an upstream regulator of several pro-inflammatory cytokines in the intestinal inflammatory response [38]. Together, these two pathways mediate downstream signaling factors via NF-κB to induce osteoclast differentiation and elicit an inflammatory response. In this study, AR495 and the >100 kDa fraction of the fermentation broth significantly inhibited the expression of these two pathways both in vivo and in vitro experiments. Therefore, it can be inferred that the >100 kDa fractions of AR495 and fermentation broth exerted anti-osteoporosis by modulating these two pathways, as well as the RANKL/RANK/OPG system. However, in in vitro experiments, we found that the unseparated fermentation broth, the >100 kDa fraction, and the 50–100 kDa fraction all significantly reduced the expression of the TLR4/MYD88 inflammatory pathway, whereas the 50–100 kDa fraction only significantly reduced the expression of the NF-κB protein, and the relative expression of the genes for the TRAF-6 protein was not significantly different from that of the model group. We speculate that this may be because the 50–100 kDa substance in the fermentation broth inhibits osteoclast differentiation mainly by affecting the inflammatory response pathway rather than intervening in bone loss by directly affecting the osteoclast formation pathway. In addition, TRAP, a glycosylated metalloproteinase-containing enzyme, is highly expressed in osteoblasts [39]. In our experiments, the activity of TRAP was reduced in the fermentation broth group, the >100 kDa group, and the 50–100 kDa group.

### 4.4. Effect of Probiotics and Other Derivatives on Disease

An increasing number of studies have identified the probiotic functions of polysaccharides, proteins, and short-chain fatty acids. Yang et al. [39] found that Poria cocos tea polysaccharides alleviated the symptoms of ulcerative colitis in mice by modulating the intestinal flora and protecting the intestinal mucosal barrier. Plant-derived polysaccharides can improve the dynamic balance of bone formation and resorption by promoting osteoblast differentiation and maturation or inhibiting osteoclast formation [40]. Short-chain fatty acids (SCFA) affect local and systemic immune function and are potent regulators of osteoclast metabolism and bone homeostasis [41]. Proteins are also closely related to osteoporosis. Xu et al. [42] investigated the association of serum and plasma proteins with bone mineral density and osteoporosis and explained these osteoporosis-related proteins, including bone aging and accelerated aging mechanisms. In our experiments, we preliminarily hypothesized that the active ingredients in the fermentation broth that exerted anti-osteoporosis were proteins and polysaccharides, which may alleviate osteoporosis by regulating bone density, bone metabolic homeostasis, and immunity [40,42].

## 5. Conclusions

Overall, this study advanced the understanding of the active components of AR495 in mitigating osteoporosis. The findings indicated that both AR495 and its fermentation supernatant exhibited enhanced anti-osteoporosis effects, with particular efficacy observed in the >100 kDa fraction of the fermentation broth. This fraction may alleviate osteoporosis by interacting with host osteoblasts, preserving the integrity of the intestinal barrier, and modulating the bone metabolic pathway. It is hypothesized that the >100 kDa fractions contain molecules that contribute to bone formation and/or inhibit osteoclastogenesis. However, the components and specific mechanisms that mediate the bone protective function still need to be further investigated.

## Figures and Tables

**Table 1:** Dosage for different treatment groups.

Groups	Dosage (Gavage)
Control	sterilized water (10 mL/kg per day)
Model	sterilized water (10 mL/kg per day)
Live	1 × 10^9^ CFU/mL (10 mL/kg per day)
Die	1 × 10^9^ CFU/mL bacteria (10 mL/kg per day)
Intra-cellular	10 mL/kg per day
Cell wall	10 mL/kg per day
Fermented	10 mL/kg per day
MRs	10 mL/kg per day
E2	10 mg/kg per day

**Table 2:** Primer sequences for quantitative real-time polymerase chain reaction.

Gene	Forward Primer (5′-3′) Reverse Primer (5′-3′)
HPRT1	GAAGGAGATGGGAGGCAATCACATT AATCCAGCAGGTCAGCAAAGAACTT
Claudin-3	TCATCGGCAGCAGCATCATCAC CCAGCAGCGAGTCGTACATCTTG
E-cadherin 1	CAGGTCTCCTCATGGCTTTGC CTTCCGAAAAGAAGGCTGTCC
Occludin	TTGAAAGTCCACCTCCTTACAGA CCGGATAAAAAGAGTACGCTGG
TLR4	CCGCTCTGGCATCATCTTCA CCCACTCGAGGTAGGTGTTTCTG
TNF-α	AGGGTCTGGGCCATAGAACT CCACCACGCTCTTCTGTCTAC
IL-6	GAGGATACCACTCCCAACAGACC AAGTGCATCATCGTTGTTCATACA
IL-1β MYD88 NF-κB RANK RANKL OPG IL-17 ZO-1 JAM-3 TRAF-6	GAAATGCCACCTTTTGACAGTG TGGATGCTCTCATCAGGACAG GCATGGTGGTGGTTGTTTCTG GAATCAGTCGCTTCTGTTGG ACACTGGAAGCACGGATGAC TGTCTGTGAGTTGCCGGTCT TGAGCCTCCGAGCAGAACTGAC TGCCTGTGTAGCCATCTGTTGAGT GATGGAAGGCTCATGGTTGGATGTG GGCAGCATTGATGGTGAGGTGTG GCAGAGACGCACCTAGCACTGA CGCAGCACAGCCACTTGTTCAT TTTAACTCCCTTGGCGCAAAA CTTTCCCTCCGCATTGACAC GCCGCTAAGAGCACAGCAA TCCCCACTCTGAAAATGAGGA CTGCGACTTCGACTGTACG TTCGGTTGCTGGATTTGAGATT AAAGCGAGAGATTCTTTCCCTG ACTGGGGACAATTCACTAGAGC

## Data Availability

The original contributions presented in the study are included in the article and Supplementary Materials; further inquiries can be directed to the corresponding authors.

## Supplementary Materials

The following supporting information can be downloaded at: https://www.mdpi.com/article/10.3390/foods13193115/s1, Table S1: Effect of different molecular weight fractions on the viability of RAW264.7 cells; Table S2: Co-culture osteoclast counts of RAW264.7 with AR495 different molecular weight fractions.
