# Supplementary material for: Evaluation of Functional Components of Lactobacillus plantarum AR495 on Ovariectomy-Induced Osteoporosis in Mice And RAW264.7 Cells
Source: Foods. 2024 Sep 29;13(19):3115. doi: 10.3390/foods13193115 (PMC11476097; doi:10.3390/foods13193115)
Supplement: Supplementary file 1 [file foods-13-03115-s001.zip › foods-3174193-supplementary.pdf]

**Supplementary Tables**

**Table S1.** Effect of different molecular weight fractions on the viability of RAW264.7 cells

| Groups | Relative cell viability (%) |
| --- | --- |
| Control | 100±0 |
| Model | 92.09±0.40$^{bc}$ |
| Fermented | 52.77±1.44$^{a}$ |
| > 100KDa | 98.25±0.62$^{c}$ |
| 50-100KDa | 92.05±6.19b$^{c}$ |
| 10-50KDa | 84.20±1.63$^{b}$ |
| < 10KDa | 47.33±1.38$^{a}$ |

All data are expressed as mean ± SD and analyzed by one-way ANOVA Duncan's multiple comparison test, n= 10. Significant differences ($P < 0.05$) are indicated with different letters (a, b, and c).

**Table S2.** Co-culture osteoclast counts of RAW264.7 with AR495 different molecular weight fractions

| Groups | Cell count (pcs) |
| --- | --- |
| Model | 15.08±0.18$^{e}$ |
| Fermented | 6.83±0.42$^{a}$ |
| > 100KDa | 6.04±0.22$^{b}$ |
| 50-100KDa | 9.19±0.31b$^{c}$ |
| 10-50KDa | 13.83±0.44$^{d}$ |
| < 10KDa | 15.17±0.08$^{e}$ |

All data are expressed as mean ± SD and analyzed by one-way ANOVA Duncan's multiple comparison test, n= 10. Significant differences ($P < 0.05$) are indicated with different letters (a, b, and c).
